# Analysis of time delay between computed tomography and digital subtraction angiography on the technical success of interventional embolisation for treatment of lower gastrointestinal bleeding

**DOI:** 10.1002/jmrs.373

**Published:** 2019-12-30

**Authors:** Gillian Bruce, Brendan Erskine

**Affiliations:** ^1^ Department of Radiology Alfred Health Melbourne Victoria Australia

**Keywords:** Computed tomography, digital subtraction angiography, embolisation, interventional radiology, lower gastrointestinal bleeding, positive correlation, technical success, time delays

## Abstract

**Introduction:**

A retrospective study was undertaken to determine a potential relationship, based on the time delay, between a positive lower gastrointestinal bleed demonstrated on computed tomography (CT) and a positive digital subtraction angiographic (DSA) study and the impact on technical success.

**Methods:**

This study investigated the correlation of time delays between imaging modalities and technical success with endovascular embolisation procedures over a 10‐year period.

**Results:**

A total of 110 patient events were analysed, and it was observed that the greater the time delay between modalities (up to 7 h), the weaker the correlation between a bleed observed on CT and DSA. This was also reflected by the technical success of the embolisation treatment. Patients experienced shorter delays when the event occurred out of normal business hours, however with decreased rates of technical success.

**Conclusions:**

There is a suggestion patients should be escalated to the angiography suite for DSA imaging as soon as possible to maximise the ability to angiographically observe acute bleeding and treat appropriately with interventional embolisation. More research in this area is required to statistically confirm this.

## Graphical abstract

A retrospective study was undertaken to determine a potential relationship, based on the time delay, between a positive lower gastrointestinal bleed (LGIB) demonstrated on computed tomography (CT) and a positive digital subtraction angiographic (DSA) study and the impact on technical success. There is a suggestion from the data that patients should be escalated to the angiography imaging suite as soon as possible to maximise the ability to angiographically observe acute bleeding and treat appropriately.

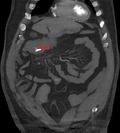

## Introduction

Acute bleeding in the lower gastrointestinal tract is a serious and life‐threatening situation that can lead to reported mortality rates of around 10%.^1^, ^2^, ^3^, ^4^ Lower gastrointestinal bleeding (LGIB) is defined as bleeding that occurs distal to the ligament of Treitz and can originate arterially from the superior mesenteric artery (SMA), inferior mesenteric artery (IMA) or internal iliac arteries.^1^, ^5^, ^6^, ^7^ LGIB can also have venous sources that are attributed to bleeding haemorrhoids involving the internal or external rectal venous plexus.^5^ LGIB predominately affects the elderly population and is commonly caused by diverticular disease, angiodysplasias, post‐polypectomy bleeding, ischaemic colitis and neoplasms.^1^, ^3^, ^5^, ^7^, ^8^ Up to 85% of patients requiring hospitalisation can be managed conservatively with sedation, bed rest and replacement of blood volume with the remainder requiring interventional treatments.^5^, ^6^, ^7^, ^9^, ^10^, ^11^ Acute LGIB is dynamic, leading to limitations with all treatment strategies, and is often associated with poor outcomes irrespective of multidisciplinary treatments.^12^, ^13^

Various imaging investigations are employed to diagnose and define a source of bleeding including colonoscopy, nuclear scintigraphy with technetium‐99m‐labelled red blood cell (RBC) or Tc‐99m sulphur colloid, computed tomography (CT) and transcatheter digital subtraction angiography (DSA).^1^, ^3^, ^5^, ^7^, ^14^ Due to the varying limitations of colonoscopy and scintigraphy, CT is used primarily as the diagnostic tool of choice where high sensitivity aids in the depiction of arterial anatomy to facilitate accurate catheterisation.^1^, ^3^, ^7^, ^8^, ^15^ DSA requires minimal patient preparation and can be performed despite large amounts of blood in the GI tract with bleeding localisation rates of up to 70%.^1^, ^8^, ^9^, ^15^ It provides the opportunity for immediate interventional transcatheter embolisation treatment with published overall technical success rates of up to 100%^8^, ^16^ and acceptable rates of complications.^15^, ^17^ This success, however, relies on the visualisation of active bleeding with reported rates of negative angiograms in up to 52% of cases.^18^, ^19^

Digital subtraction angiography‐guided interventional embolisation appears to be most useful in patients who do not respond to conservative treatment, those with high surgical risks or patients unlikely to tolerate bowel preparation and urgent colonoscopy.^8^, ^16^ Treatment decisions for patients with LGIB are widely varied in the literature and are often based on local expertise and preference.^3^, ^12^ For patients experiencing acute LGIB, our institution commonly facilitates an abdominal CT followed by imaging and subsequent embolisation treatment in an angiography suite using DSA as an efficient treatment pathway. It is assumed a shorter time delay between these modalities maximises the potential of positive imaging correlation, bleeding localisation and subsequent embolisation treatment. Whilst there is extensive literature on the benefits of angiographic embolisation techniques in the setting of emergency haemodynamically unstable active bleeding, an extensive literature review found almost no reference to the time frame in which this was performed.^13^, ^18^, ^20^ A six‐year study of 42 patients conducted by Koh et al^21^ highlighted an importance in a timely transfer of a patient between modalities and found a significant increase in detection if the DSA was performed within 90 min of the abdominal CT. In a smaller study by Tan et al,^15^ it was found that performing an invasive mesenteric angiogram within 150 min of an initial abdominal CT slightly increased the likelihood of identifying the bleeding site in angiography although this difference was not deemed statistically significant. Using a larger patient cohort, a study has been conducted to draw comparisons to these existing results with an aim to definitively determine whether time delays between modalities affect the technical success of DSA‐guided embolisation.

## Materials and Methods

### The LGIB patient

Our institution’s abdominal CT protocol for presentations clinically indicating LGIB describes a triple‐phase helical scan. This includes a preliminary non‐contrast acquisition to depict pre‐existing hyper‐dense areas that may be misinterpreted as active bleeding, an arterial acquisition (using 80 mL of contrast followed by a 40 mL saline chase injected at 4 mL/sec) to visualise intra‐luminal blushing indicative of a positive GI bleed and a delayed scan (acquired 150 sec post‐contrast injection) to capture contrast pooling that may also indicate a bleeding source. Images processed and sent to the picture archiving and communication system (PACS) system include non‐contrast axial images, axial/coronal/sagittal multiplanar reformats for arterial and delayed sequences, and coronal and sagittal maximum intensity projections (MIPs) in the arterial phase. Reformatted coronal MIPs (10‐mm slice thickness/5‐mm slice interval) are anecdotally regarded as the most beneficial to correlate imaging and plan an anatomical pathway for super‐selective DSA imaging.

In circumstances where signs suggestive of LGIB are observed on CT, and the patient is a candidate for interventional transcatheter treatment, they will be transferred to the angiography suites as soon as possible. Our institution offers a 24‐h emergency service which includes CT imaging however the interventional angiography team operates during regular business hours with an on‐call team available outside of these times. The patients may be delayed between these modalities due to the availability of the imaging resources, delays from the referring unit, the ability to gain consent, coagulopathy and patient stabilisation prior to transfer.

Although highly user‐dependent, the protocol for selective abdominal angiography at our institution describes groin access via the common femoral artery for selection of the SMA and/or IMA branches. An aortogram may be performed in circumstances where variant anatomy is present or there is difficulty engaging the vessel ostium. Contrast injections in selected vessels are achieved using a variety of 4–5 Fr catheters utilising either a power injector or hand injection by the operator (Table 1). Imaging projections are highly user‐dependent depending on area of bleeding and potentially obstructing bowel gas shadows. Respiration is withheld where possible and un‐subtracted images may also be utilised to delineate the bowel and differentiate motion artefacts. Super‐selective imaging with micro‐catheters is performed where possible, to ensure accuracy in embolisation, predominately with the use of contrast hand injections.

**Table 1:** Contrast power injector settings and imaging acquisition rates for digital subtraction angiographic of the abdomen.

Vessel of interest	Volume/rate (mL/sec)	Pressure limit (PSI)	Frame rate (fps)
Superior mesenteric artery (SMA)	30 mL @ 5 mL/sec	300	2 × 4 sec 1 × 3 sec 0.5 × 20 sec
Inferior mesenteric artery (IMA)	15 mL @ 3 mL/sec	300	
Iliac arteries	6 mL @ 3 mL/sec	300	
Super‐selective branches (microcatheter)	8 mL @ 2 mL/sec	750 (with appropriate rate rise)	

A variety of embolisation agents may be used to occlude bleeding including micro‐coils, gelfoam, glue or PVA particles. The selection of these agents is left to the discretion of the interventionalist. Although micro‐coils are most often the agent of choice, embolisation may utilise more than one technique in severely coagulopathic patients. Contraindications to embolisation include coagulopathy (INR > 3.0), inconclusive angiographic study, contrast allergy, previous surgery or radiotherapy, and severe atherosclerosis. Non‐identification of a bleeding vessel often triages patients into a group who will have a benign course. For this reason, anecdotally, our institution has not found augmentation of haemorrhage with haemolytics or vasodilators of use.

**Figure 1:**
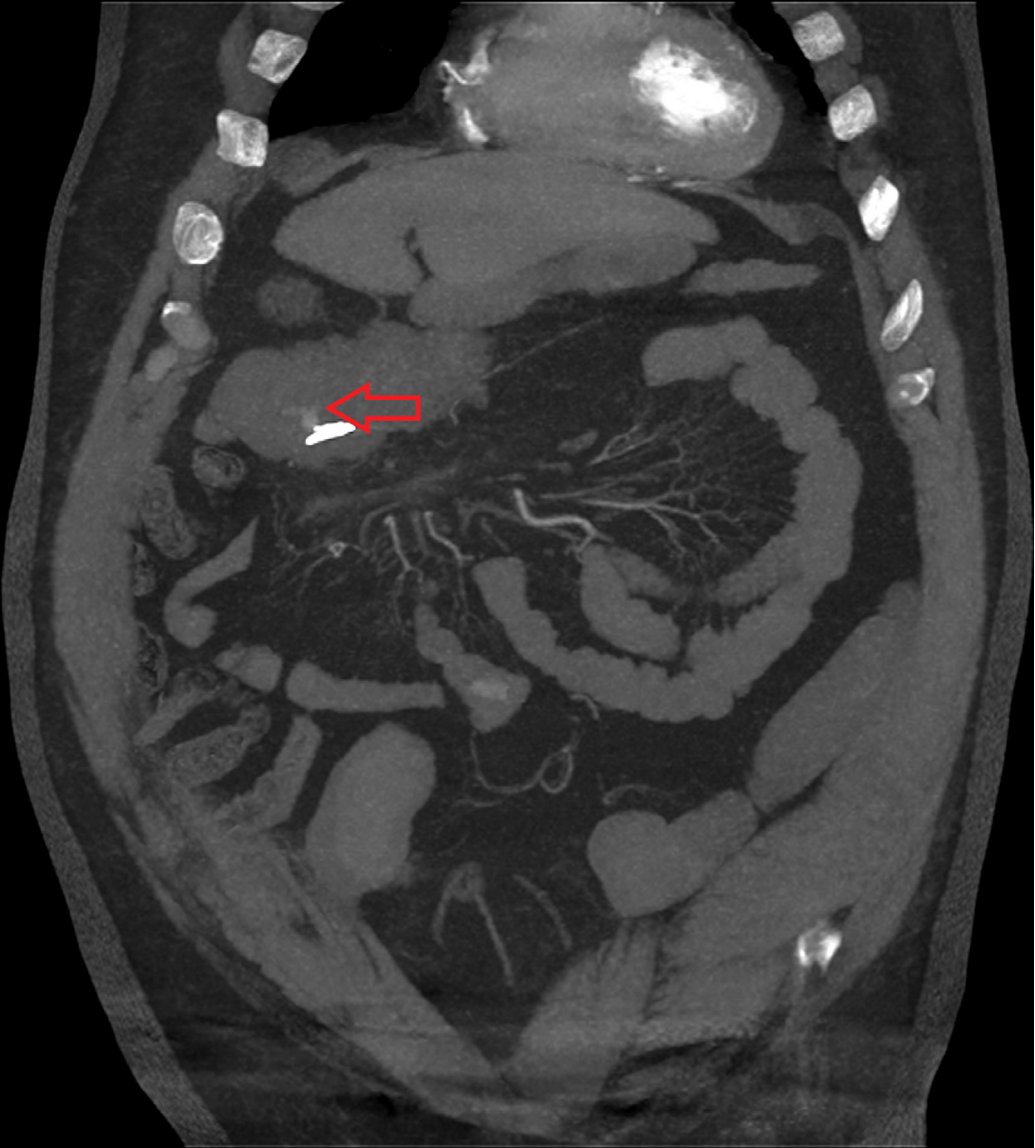
Positive correlations were defined as an active blush detected on computed tomography (CT) and an active blush observed on digital subtraction angiogram (DSA) (Figs. 1 and 2). A successful embolisation was deemed a treatment that stopped any apparent bleeding on immediate imaging (Fig. 3). This is a case of a 73‐year‐old male presenting with a large volume per rectal bleed post‐colonoscopy. A coronal MIP CT image from an arterial acquisition shows active bleeding from the transverse colon, observed in right upper quadrant.

**Figure 2:**
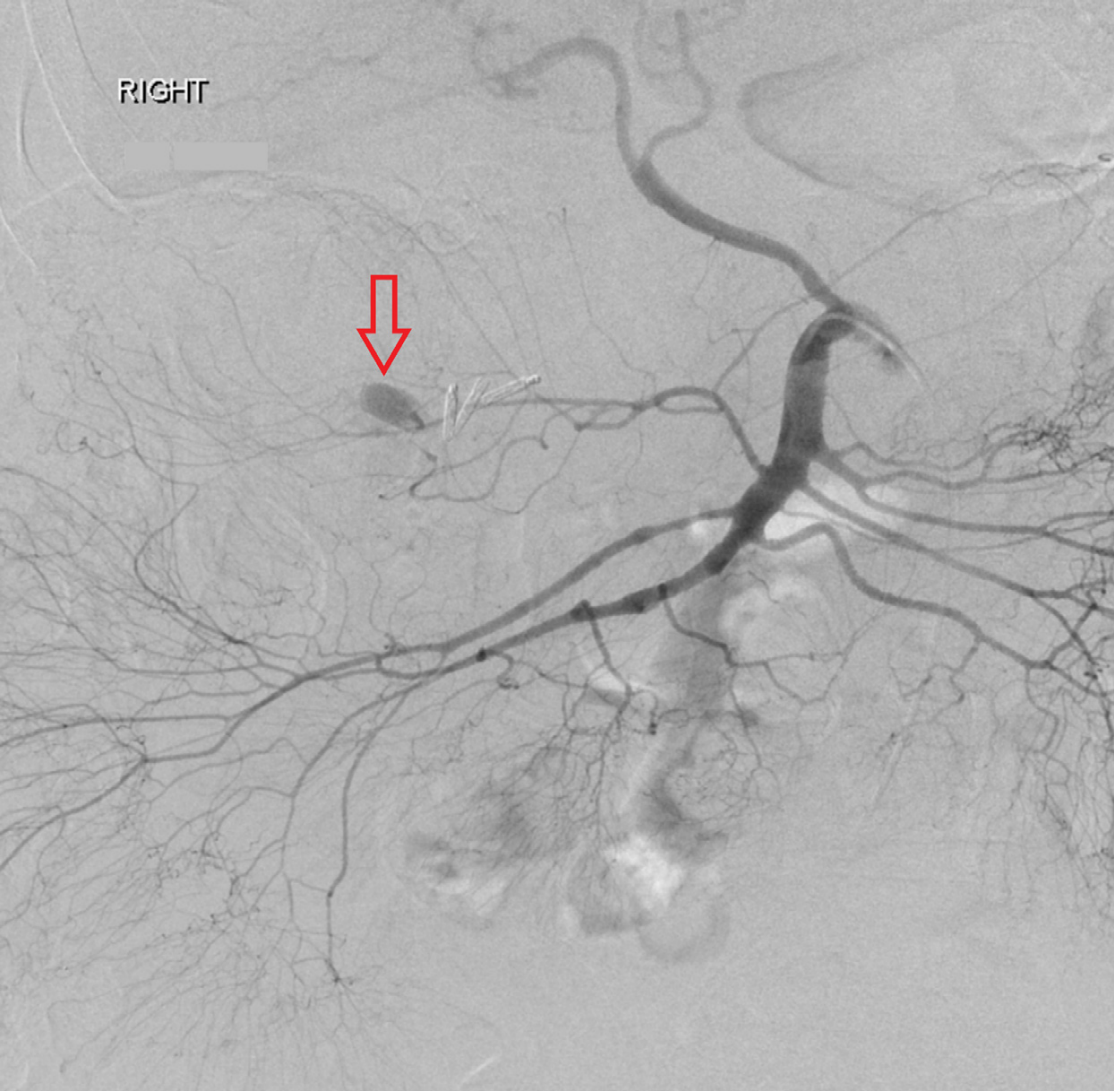
Following a 40‐min delay experienced between computed tomography (CT) and digital subtraction angiogram (DSA) imaging, the same bleed is observed on PA DSA imaging. Previous cholecystectomy clips are noted in the region of the bleed.

**Figure 3:**
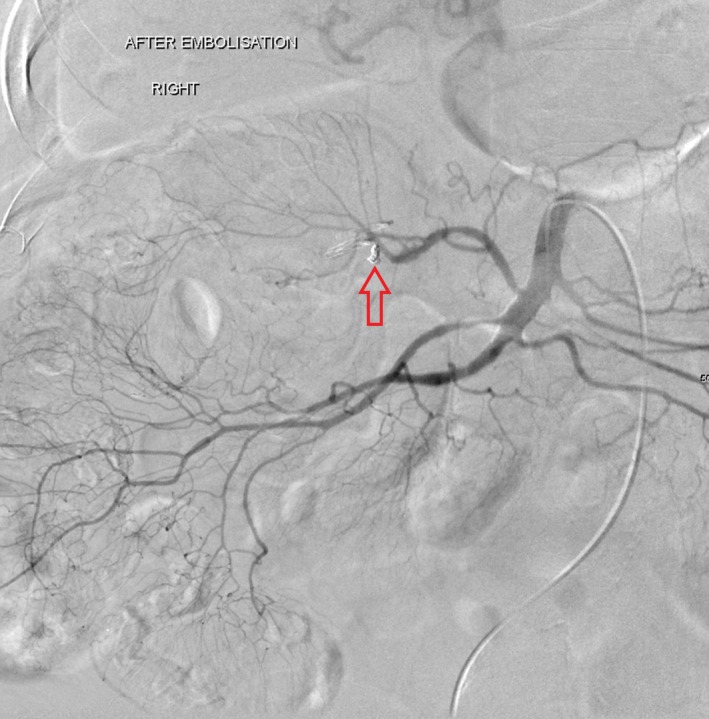
Post‐embolisation of bleed with the use of micro‐coils. No evidence of contrast blush seen following treatment indicating a technically successful patient event.

Following ethics approval from our institutional review board, a retrospective de‐identified data analysis was conducted in accordance with current ethical standards. This was inclusive of all patients between January 2007 and December 2017 with acute LGIB that underwent a multi‐slice contrast‐enhanced abdominal CT followed by DSA imaging. This was performed using the digital imaging and communications (DICOM) information on PACS and the associated radiology reports. Patients following this treatment pathway on multiple occasions were deemed to be separate events. For each patient event, the time of the last axial slice of the multi‐slice CT in the arterial phase was acquired and the time of the first DSA image was recorded. This decision was made as not all patients underwent a CT delayed phase of imaging and provided a consistent timestamp for patient comparison. The lack of triple‐phase CT imaging is assumed to be due to patient limitations to complete the study, radiographer error or LGIB detected as an incidental finding on an arterial scan. The patient demographics, suspected pathology, bleeding vessel origin and embolisation agents were also documented. Technical success was defined as a positive correlation between CT and DSA imaging which was then successfully treated with embolisation. A successful embolisation was deemed a treatment that stopped any apparent bleeding on immediate imaging. Patients in whom the bleed was not observed on DSA and no treatment administered were classified as treatment not required. Patients were divided into time delay groups of one hour as well as grouped into those that were imaged during and outside of working business hours. Patients were also collated into either positive or negative correlation groups. Positive correlations were defined as an active blush detected on CT and an active blush observed on digital subtraction angiogram (DSA) (Figs. 1, 2, 3). Negative correlations were defined as a positive blush detected on only one modality of imaging, either CT or DSA. Data were analysed using SAS software version 9.4 (SAS Institute, Cary, NC, USA). Univariate analysis was used for categorical independent variable (time categories) and Fisher’s exact test to determine two‐sided *P*‐values (significance of 0.05). Positive and negative correlations were reported as percentages.

## Results

This retrospective study collated a total of 110 patients who had undergone a contrast‐enhanced abdominal CT followed by a selective abdominal DSA investigation. There were a total of 36 females and 74 males ranging in age from 21 to 101 years (median = 78). The majority of bleeds observed originated from the IMA (*n* = 58; 52.7%) closely followed by the SMA (*n* = 46; 41.8%) with a small percentage from a combination of the IMA and SMA (*n* = 4; 3.6%) and internal iliac arteries (*n* = 2; 1.8%). Across the total patient cohort, there were three patients who despite a negative CT went on to have a positive angiographic investigation because of clinical indication. One referral was for a massive rectal bleed and another with a prior positive nuclear medicine scan in addition to continued bleeding. It is assumed in the third case the patient was clinically indicated for further imaging and a repeat CT was bypassed. In all cases, the patients were embolised.

Time delays recorded between the two modalities ranged from a minimum of 32 min to a maximum of 915 min (median = 158 min).

A total of 75 patients (68.2%) underwent embolisation treatments. Comparisons over time are shown in Table 2 with the highest rate of embolisation occurring in those patients seen within 60 min of CT imaging followed by those in the 60 to 119 min delay time frame.

**Table 2:** Time delays experienced between computed tomography and digital subtraction angiographic modalities, imaging correlation and resulting embolization.

Time delay (min)	Total patients *(n)*	Positive correlation *(n)*	Positive and embolised Technical success *(n,* %)	Negative correlation Treatment not required *(n,* %)	Negative and embolised *(n,* %)	Total embolised *(n,* %)	*P*‐value (increasing time)
<59	5	4	4 (80.0)	1 (20.0)	0 (0.0)	4 (80.0)	X
60–119	18	14	13 (72.2)	4 (22.2)	1 (5.6)	14 (77.8)	1
120–179	47	29	28 (59.6)	18 (38.3)	4 (8.5)	32 (68.1)	0.64
180–239	21	12	11 (52.4)	9 (42.9)	2 (9.5)	13 (61.9)	0.62
240–299	10	7	7 (70.0)	3 (30.0)	0 (0.0)	7 (70.0)	1
300–359	3	2	2 (66.7)	1 (33.3)	0 (0.0)	2 (66.7)	1
360–419	3	0	0 (0.0)	3 (100.0)	1 (33.3)	1 (33.3)	1
420+	3	3	2 (66.7)	0 (0.0)	0 (0.0)	2 (66.7)	1
Total	110	71	67	39	8	75	

Embolisation agents included micro‐coils (*n* = 56; 74.7%), gelfoam (*n* = 7; 9.3%) and PVA particles (*n* = 4; 5.3%). In a small number of cases, more than one embolic agent was used including PVA particles and micro‐coils (*n* = 4; 5.3%), micro‐coils and gelfoam (*n* = 3; 4.0%) and a single case of gelfoam and PVA particles (*n* = 1; 1.3%).

Of those 71 patients with a positive correlation between CT and DSA, four patients were unable to be treated using embolisation. This is attributed in the associated reports to be due to factors including vessel dissection (*n* = 1), vasospasm (*n* = 2) and a tortuous vessel origin that was unable to be cannulated (*n* = 1). In addition, there were four patients who following a positive CT had a negative DSA (negative correlation) however underwent embolisation (prophylactic treatment).

**Figure 4:**
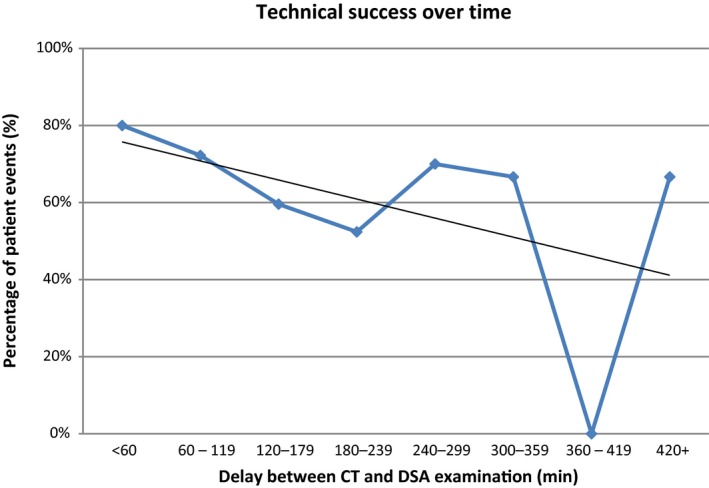
Technical success over time experienced over increasing time delays between computed tomography (CT) and digital subtraction angiogram (DSA) examinations.

The highest rate of technical success was found in patients who were imaged using DSA within 60 min of the CT scan; however, no significant difference in the technical success was found across the different time points (*P* = 0.56) (Fig. 4). The overall technical success rate had a *P*‐value = 0.56. All *P*‐values were >0.05 (see Table 2).

When evaluating the time of day patients underwent DSA imaging, it was found a total of 38 (*n* = 38, 34.5%) candidates were imaged within normal working hours (0830–1700 h) with a median time delay of 176 min. 27 (*n* = 27, 71.1%) of these patients had a positive correlation between their CT and DSA imaging all undergoing embolisation treatment. This population had a technical success rate of 65.8% (*n* = 25). The remaining 72 patients were imaged out of hours (1700–0830 h) with a median time delay of 155 min. 44 (*n* = 44, 61.1%) had a positive imaging correlation with 48 undergoing embolisation. In this population, there were 42 technically successful events (*n* = 42, 58.3%).

When reviewing the associated CT reports, the vast majority did not describe a cause of bleeding (*n* = 58; 52.7%). The most common pathology described was a bleeding diverticula (*n* = 36; 32.7%) followed by angiodysplasia (*n* = 8; 7.2%), post‐operative bleeding (*n* = 2; 1.8%), pseudoaneurysm (*n* = 2; 1.8%) and single cases of actively bleeding enteritis (*n* = 1) and neoplasm (*n* = 1). Despite being a large trauma hospital network, only two cases of LGIB bleeding from a traumatic source were documented (*n* = 2). Although not standard practice, there were three instances where provocative bleeding was induced in the DSA suites through the use of glyceryl trinitrate (GTN) as a vasodilating agent. This was facilitated when there was a strong clinical suspicion for LGIB despite a negative CT, and it was hoped this would be visible on angiographic imaging. In these cases, there continued to be no evidence of bleeding on the DSA images and subsequently no embolisation performed.

## Discussion

LGIB in the acute setting is a serious condition which requires timely imaging and treatment. CT is a widely accepted, readily available imaging modality with high sensitivity in detecting LGIB. Due to the immediate treatment ability using embolisation techniques, DSA of the abdomen may be an advantageous modality for the management of patients bleeding from the lower GI tract. Together these modalities can create a beneficial treatment pathway for the acute patient. Negative results in both settings may be common due to the potential intermittent nature of a bleeding source, so there is a need for fast and accurate detection and localisation.^14^, ^19^ This study aimed to draw comparisons with existing research and provide further evidence of the most beneficial timeframe in which patients should be transferred to the angiography suites for treatment following a positive CT scan for LGIB.

At our institution, delays in treatment have been observed to be caused by a variety of factors including the availability of reporting services at the time of CT imaging to escalate efficiently to the treating clinicians, the experience of the reporting radiologist or the quality of CT imaging (e.g. patient motion artefacts). Delays may also be experienced as angiography resources available vary throughout the day due to a triaging of acute patients, procedures in progress at the time of diagnosis or events occurring out of business hours requiring an ‘on‐call team’ to attend. There is also potential for delays from the referring unit when time is taken to decide on the best pathway of treatment, an attempt at conservative management or due to patient limitations. Examples include contrast allergies discovered from the initial CT scan, insufficient renal function, coagulopathy, ability to obtain consent and haemodynamic stabilisation before being transported to the interventional suite.

The small sample size may have contributed to the results not being statistically significant; however, several trends were observed. One observation of interest related to the rates of technical success over time. It was found that the highest rate of technical success occurred in patients investigated with DSA in under one hour, closely followed by those imaged between one and 2 h. Following on from this, it was assumed this would decline in a linear fashion; however, after steadily decreasing, the rate of technical success spiked again between 4 and 5 h delay and after 8 h delay. This describes a suggestion of the significance of escalating acute LGIB patients to the angiography suites as soon as possible. In addition, there appears to also be potential in treating patients using DSA guidance even if a substantial delay has occurred following a positive CT scan (such as those transferred from another hospital).

The lowest rates of embolisation treatment occurred between the 7‐ and 8‐h delays between modalities. This could be attributed to those patients in whom the bleeding ceased organically. After 8 h, this rate begins to rise again, although these data have been taken from a small number of patient events. Potentially, these patients began another episode of active bleeding and raise the question of whether there should be more of a ‘wait‐and‐see approach’ for patients falling in the 7‐ to 8‐h time delay periods who are haemodynamically stable.

There were five patients that, despite being unable to see the bleed on DSA imaging, underwent prophylactic treatment. In order to embolise a vessel where the origin of bleeding cannot be observed requires a supreme level of diagnostic confidence. All but one of these cases occurred during 2015 and is assumed to be due to operator bias.

A proportion of patients had a positive correlation between CT and DSA however were unable to be embolised due to other factors (vessel spasm, inability to adequately cannulate the feeding vessel or dissection). This in turn cannot always be seen as a failure to treat. In cases which experienced vessel spasm, as an example, the active bleed may then be temporarily stopped and as a result the point of bleeding spontaneously thrombose.

As patients with acute LGIB present in an unpredictable nature, all interventions were undertaken on an ‘emergency basis’. During business working hours (weekdays 0830–1700 h) DSA investigations would have been performed as soon as possible; however, if required out of hours, they would demand the assembly of a skeleton ‘on‐call’ team. Those seen within hours had a higher rate of positive correlation between imaging modalities and technical success despite longer median time delays. It is proposed that these advantageous results may be due to the increased abilities of well‐rested staff and their potential to persist in localisation of a bleeding source in addition to a greater pool of resources and knowledge from surrounding colleagues. In lieu of the findings above, patients presenting with acute LGIB overnight could potentially be suitable to wait until the following morning for their interventional procedure. This may increase their chances of achieving technical success however would only be a consideration in the presence of haemodynamic stability.

When reviewing the methods of embolisation, over 84% of treatments utilised micro‐coils, consistent over the 10‐year study. Micro‐coils are a permanent method of embolisation with high success rate, however their use limits the ability to re‐enter a vessel in cases of a re‐bleeding. This is in accordance with the study by Keeling et al^16^ who also found coils to be the most commonly used agent in the embolisation of LGIB. From this study provocative angiography was found to be utilised in only 2.7% of cases. This is particularly small in contrast to the results of Bloomfield et al^22^ who describe a diagnostic yield of 29% following provocative angiograms.

## Study Limitations

In this study, the level of operator bias involved was difficult to distinguish as the level of experience of the CT reporting radiologists was not recorded nor was the skill level of the interventional radiologist performing the DSA investigation. It is also recognised that there may have been a selection bias for patients nominated to DSA. Unlike other studies performed, patient haemodynamic status was not assessed. It is hoped this study may be used as a basis for further investigation into this element of patient presentation and associated patient outcomes.

The images associated with the study in both CT and DSA were performed on a range of equipment over the 10‐year period of collection due to system updates and new machine installations. Those in emergency were scanned using a GE 64 slice VCT LightSpeed (01/01/2007‐22/5/2014) or Canon 320 slice Aquilion 1 (23/05/2014‐31/12/2017). Inpatients were scanned using a GE 16 slice LightSpeed PRO (01/01/2007–05/06/2013) before a GE 128 slice Discovery 750 HD (05/06/2013‐31/12/2017). The scanner used for each patient was not compared in this study however could be an area for further investigation.

Despite verification, human error is also recognised as a limitation in this study as reports were accessed and entered into a database manually. Treatment pathways for patients are made on an individual basis, and the related discussions between clinicians are not included in their associated imaging reports to be analysed and compared. Details of procedural techniques and potential delays were collated following discussions with clinicians and interventional radiologists on site and are subject to personal interpretations.

## Conclusion

Ten‐year data were collected to investigate patients who underwent DSA following contrast‐enhanced abdominal CT positive for acute LGIB. We observed that increasing time delay between modalities reduced the detection rate of bleeding on DSA and overall technical success with transcatheter embolisation; however, this did not reach statistical significance.

Whilst the highest technical success was achieved when patients were imaged with DSA within 60 min of their CT, technical success rates did not sharply drop in the hours following, suggesting that even with significant delays there is still benefit in investigating the bleed further in angiography. At what point this technique is no longer of any benefit is an area difficult to assess as standard protocol is to assess the patient as promptly as possible.

A trend was observed in patients who were investigated using DSA during normal working hours having a better outcome when comparing technical success. This should be a point of consideration when formulating an individual treatment plan for the acute LGIB patient.

This study includes information that can potentially be used to highlight the need for priority transfer of patients with active LGIB to optimise technical success. This study and further larger studies may contribute to the establishment of protocols and guidelines of a time‐based treatment pathway for patients with LGIB.

## Funding Information

This manuscript was not supported by any funding.

## Conflict of Interest

The author declares no conflict of interest.
